# Effects of social network incentives and financial incentives on physical activity and social capital among older women: a randomized controlled trial

**DOI:** 10.1186/s12889-021-10175-3

**Published:** 2021-01-21

**Authors:** Ryo Yamashita, Shinji Sato, Ryoichi Akase, Tatsuo Doi, Shigeki Tsuzuku, Toyohiko Yokoi, Shingo Otsuki, Eisaku Harada

**Affiliations:** 1 Kumamoto Institute of Total Fitness, 6-8-1 Yamamuro, Kita-ku, Kumamoto, 860-8518 Japan; 2 Teikyo Heisei University, 2-51-4 Higashiikebukuro, Toyosima-ku, Tokyo, 170-8445 Japan; 3 Kumamoto Kinoh Hospital, 6-8-1 Yamamuro, Kita-ku, Kumamoto, 860-8518 Japan; 4 Dynamic Sports Medicine Institute, 1-10-28 Nishishinsaibashi, Chuo-ku, Osaka, 542-0086 Japan; 5 Kumamoto University, 2-39-1 Kurokami, Chuo-ku, Kumamoto, 860-8555 Japan; 6 Osaka Sangyo University, 3-1-1 Nakagaito, Dito-city, Osaka, 574-8530 Japan

**Keywords:** Older women, Social network incentive, Financial incentive, Physical activity, Social capital

## Abstract

**Background:**

Financial incentives have been used to increase physical activity. However, the benefit of financial incentives is lost when an intervention ends. Thus, for this study, we combined social network incentives that leverage the power of peer pressure with financial incentives. Few reports have examined the impact of physical activity on social capital. Therefore, the main goal of this study was to ascertain whether a combination of two incentives could lead to more significant changes in physical activity and social capital during and after an intervention.

**Methods:**

The participants were 39 older women over 65 years of age in Kumamoto, Japan. The participants were randomly divided into a financial incentive group (FI group) and a social network incentive plus financial incentive group (SNI + FI group). Both groups underwent a three-month intervention. Measurements of physical activity and social capital were performed before and after the intervention. Additionally, the effects of the incentives on physical activity and social capital maintenance were measured 6 months postintervention. The financial incentive group received a payment ranging from US$4.40 to US$6.20 per month, depending on the number of steps taken during the intervention. For the other group, we provided a social network incentive in addition to the financial incentive. The SNI + FI group walked in groups of three people to use the power of peer pressure.

**Results:**

A two-way ANOVA revealed that in terms of physical activity, there was a statistically significant interaction between group and time (*p* = 0.017). The FI group showed no statistically significant improvement in physical activity during the observation period. In terms of the value of social capital, there was no significant interaction between group and time.

**Conclusion:**

Our results suggest that social network incentives, in combination with financial incentives, are more effective for promoting physical activity than financial incentives alone among older women and that these effects can continue after an intervention. In the meantime, further studies should be conducted on the effect of physical activity on social capital.

**Trial registration:**

UMIN000038080, registered on 09/22/2019 (Retrospectively registered).

## Background

It is common knowledge that an increase in physical activity improves health. It reduces the risk of mortality and cardiovascular diseases [1–4], improves physical function [5], and improves quality of life [6, 7]. Additionally, the preventive effects of cognitive impairment [8, 9], depression [10, 11], and cancer [12, 13] have been reported recently. Despite these beneficial effects, the level of physical activity is still low among the population [14, 15].

Previous studies have shown that financial incentives have been used to achieve behavioral change in physical activity in an effort to solve this problem [16, 17]. However, the benefit of the incentive is lost when the intervention ends [18, 19], meaning that the effect of financial incentives is short-term. Therefore, in this study, we focused on social network incentives that leverage the power of peer pressure to regulate behavior [20]. An advanced study by Aharony et al. [21] divided 108 active young people into three groups: i) a control group, ii) a peer-view group, and iii) a peer-reward group; participants were rewarded according to their increase in physical activity. In the control group, individuals were rewarded for their own increased physical activity [21]. In the peer-view group, the participant was shown his/her buddies’ activity levels, but was still rewarded for his/her own activity level. In the peer-reward group, the buddies received a reward proportional to the participant’s activity level. As a result, the peer-reward group yielded a significantly larger increase in activity level than both the control and peer-view groups. In their study, creating peer pressure by adding two “buddies” to one person was necessary to achieve this benefit. Aharony et al. called this strategy “social network incentives.” Furthermore, their study found that increased physical activity continues after the intervention has ended when financial incentives are combined with social network incentives. Therefore, combining social network incentives with financial incentives for increased physical activity may have a long-term effect. However, the participants in Aharony et al.’s study only included young people. In addition, their study did not explicitly present a randomized controlled trial. Thus, we hypothesized that the combination of social network incentives and financial incentives would have a long-term effect on physical activity in older persons, and decided to verify this using a randomized controlled trial.

Furthermore, Brach et al.’s [22] study demonstrated that physical activity plays a significant role in maintaining functional fitness in older women [22]. Additionally, a decrease in muscle strength and mass is likely to be a consequence of more physical inactivity. Therefore, it is important that older women increase their physical activity [23].

On the other hand, recent public health research revealed that individual health behavior was affected by social capital [24, 25]. This mechanism works in such a way that being integrated into a particular network means that people are under the influence of others in the same network, and as a result their individual health behavior is controlled [26]. Meanwhile, there have been no previous findings which showed that physical activity enhances individual-level social capital. Therefore, we assumed that encouraging walking in small groups using social network incentives should enhance individual-level social capital.

The main goal of this study was to ascertain whether combining financial incentives with social network incentives could lead to more significant changes in physical activity and social capital among older women compared to financial incentives alone, during and after an intervention.

## Methods

### Study design and participants

This study was a randomized study in which participants were recruited by handing out leaflets in several different regions in Kumamoto, Japan. Forty-four older women over 65 years of age were recruited between August 2017 and September 2017.

After completing measurements before the intervention, participants drew a sealed envelope to determine whether they were allocated to the financial incentive group (FI group) or the FI plus social network incentive group (SNI + FI group).

**Fig. 1:**
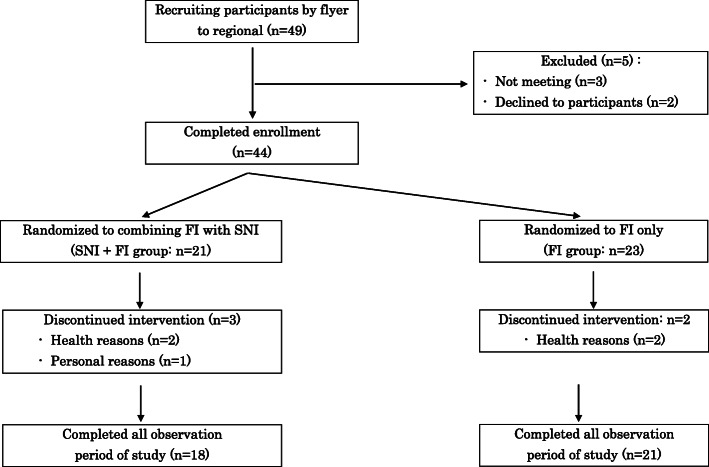
Flow diagram of the two groups’ progress through the phases the randomized trial

During the intervention, two participants in the FI group and three in the SNI + FI group dropped out. Eventually, the study groups comprised 21 participants in the FI group and 18 participants in the SNI + FI group. The study flowchart is presented in Fig. 1.

Each participant in the FI group received a payment ranging from US$4.40 to US$6.20 per month, depending on the number of steps taken per day during the intervention as follows:

(a) A $4.40 gift card if the average daily steps for a month was between 5000 and 7999 steps/day.

(b) A $6.20 gift card if the average daily steps for a month were 8000 steps/day or more.

The SNI + FI group participants walked in groups of three people to utilize the power of peer pressure, in addition to receiving the financial incentive. Three people from the SNI + FI group selected the groups in the order in which they opened the envelopes, after grouping by randomization. Consequently, most groups did not know each other in advance. The groups of buddies remained the same over the three-month intervention in this study. The participants were informed that they would walk in groups of three people about once a week. Furthermore, their rewards were designed to reflect the largest number of steps taken among their “buddies.” The buddy’s overall step count results were communicated between themselves as they walked together. In addition, regarding the reflected rewards, the number of steps taken by all group members, the reflected number of steps taken, and their rewards were delivered to each group member once a month. The payment rewards ranged from US$4.40 to US$6.20 per month and were available for both groups. After the three-month intervention, the participants were not asked to walk with their peers. However, they could decide for themselves whether to walk or not. In the SNI + FI group, if it became impossible even one in three people to walk together for whatever reason, the research was excluded.

Both groups underwent a three-month intervention between September 2017 and December 2017. Before the intervention, each group was assessed for age, body height, body weight, body mass index, and percentage of body fat. Measurements of physical activity and social capital were performed before and after each intervention. Additionally, the effects of the incentives on activity maintenance were measured 6 months after the intervention. The study design described above has followed the one in our previous study [27].

All participants provided written informed consent to participate in the study, which was approved by the ethics committee of Osaka Sangyo University (2017-JINRIN-016).

### Blinding

Those assessing the outcomes were blinded to the grouping allocation; however, owing to the nature of the intervention, participants were not blind to their allocation. The FI group participants did not know about the SNI + FI group reward structure.

## Anthropometric measures

Body height was measured to the nearest 0.1 cm. Body weight was measured to the nearest 0.1 kg using a digital scale. Body mass index (BMI) was calculated using the formula BMI = body mass (kg)/ (body height [m])^2^. The percentage of body fat was calculated using the formula: adult body fat% = (1.20 × BMI) + (0.23 × age) – (10.8 × sex)-5.4 [28].

### Physical activity

Before starting the study, a pedometer (EX-500, YAMASA TOKEI KEIKI CO., LTD, Tokyo, Japan) was given to each participant to measure the number of steps per day. Each participant also received a diary to record their daily step count (pedometer). The SNI + FI group kept record in the diary when three people walked together. For the evaluation of the number of steps, the average daily step count for 1 month was calculated based on the number of steps written in the diary. To manage the accuracy of the total number of steps, researchers collated the pedometer’s weekly data when participants submitted their diaries.

### Social capital

The measurement of social capital used trust, network, and social participation by referring to Yang’s report [29]. Trust and network of social capital was surveyed using a questionnaire created by the Japanese Cabinet Office. The trust was assessed by a single item: “Generally speaking, would you think that most people can be trusted?” The responses were selected using a Likert scale [30]. The network used two questions. The first question was regarding “Relationship with neighbors.” This question included a four-point scale (none; would greet; would talk while standing; would consult with life concerns). The second question concerned the “Number of neighbors with whom one has a relationship.” This was also a four-point scale with the following possible answers: zero, four or fewer people, five to nineteen people, and twenty people or more.

From a previous study by the Japan Science and Technology Agency Index of Competence (JST-IC), the social participation was assessed using four items [31, 32]. The four items were as follows: (1) Participate in regional events; (2) Participate in a neighborhood association; (3) Assume a managerial position or role such as the leader in a residents’ association; and (4) Engage in charity. These items were assessed using Yes = 1/No = 2, and the points were summed. All these measurements were of undefined time frame.

For social capital, individual indexes were calculated by standardizing (calculated so that the mean was “0” and the standard deviation and variance were “1”) each item. The standardization formula was $\mathrm{Y}=\frac{\mathrm{X}-\upmu}{\upsigma}$. In this formula, X represented the data, μ the mean of X, and σ the standard deviation.

### Statistical analysis

Data were analyzed using SPSS Statistics 20.0 (IBM Corporation, Tokyo, Japan). All descriptive and statistical data are shown as the mean ± SD. An unpaired t-test was used to compare the differences in age, body height, body weight, BMI, and percentage of body fat between the FI group and SNI + FI group before the intervention. Two-way repeated-measures ANOVA was conducted to compare the effects of the intervention and the six-month postintervention physical activity and social capital between groups. Post hoc analyses were conducted using simple main effects. The partial eta squared (η 2 p) was used to assess the effects size from the ANOVA analyses. The significance level was set at *p* < 0.05.

## Results

**Fig. 2:**
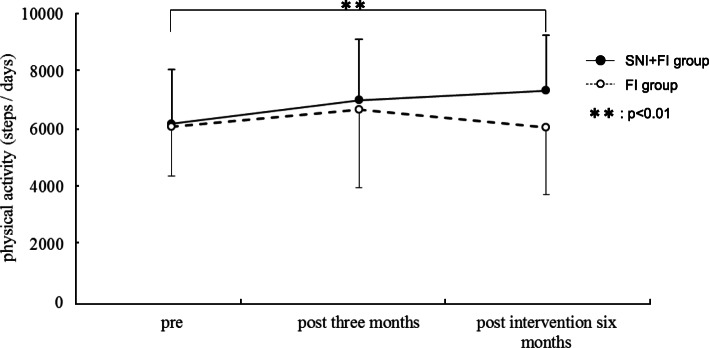
Comparison of intervention effect for physical activity

Before the intervention, there were no significant differences in age, body height, body weight, BMI, or percentage of body fat between the FI group and the SNI + FI group (Table 1). A two-way ANOVA revealed that in terms of physical activity, there was a statistically significant interaction between group and time (F (1, 37) =6.24, *p* = 0.017, Fig. 2). The physical activity of the SNI + FI group increased significantly between the preintervention and the six-month postintervention periods (F (2, 36) =5.41, *p* = 0.006, Fig. 2). However, the FI group showed no statistically significant improvement in physical activity during the observation period (Fig. 2). In terms of social capital, a two-way ANOVA revealed that, in trust, networking and the JST-IC, there was no significant interaction (trust, *p* = 0.08; network, *p* = 0.18; JST-IC, *p* = 0.84, Table 2). The social capital analysis results were the same regardless of standardization (not shown).

**Table 1:** Comparison of Clinical Characteristics between SNI + FI group and FI group

	All *n* = 39	SNI + FI group *n* = 18	FI group *n* = 21	*P*
Age (years)	71.9 ± 5.7	73.5 ± 6.3	70.6 ± 4.9	0.11
Body height (cm)	155.3 ± 6.8	153.7 ± 8.0	156.6 ± 5.5	0.19
Body weight (kg)	53.7 ± 8.7	51.3 ± 7.6	55.8 ± 9.2	0.11
Body mass index (kg/m2)	22.2 ± 2.8	21.8 ± 3.2	22.6 ± 2.4	0.36
Percentage of body fat(%)	37.3 ± 4.0	37.1 ± 5.3	37.5 ± 2.7	0.77
Trust	0.0 ± 1.0	−0.2 ± 1.1	0.2 ± 1.0	0.18
Network	0.0 ± 1.9	−0.5 ± 1.6	0.4 ± 2.0	0.12
Social participation	−0.2 ± 0.9	0.0 ± 1.0	−0.4 ± 0.8	0.12

Values are expressed as the mean ± SD

**Table 2:** Comparison of intervention effects for physical activity and social capital variable

	e	SNI + FI group			FI grou p			Interaction
	pr	post 3 months	post intervention 6 months	pr e	post 3 months	post interventi on 6 months	main effect of time	
Physical activity (steps/day)	6194.7 ± 1879.6	7014.6 ± 2098.6	7353.5 ± 1908.2**	6102.9 ± 1728.6	6696.0 ± 2714.6	6073.4 ± 2335.5	0.02	0.02
Trust	0.2 ± 1.1	0.2 ± 0.9	0.2 ± 0.8	0.2 ± 1.0	−0.1 ± 1.1	− 0.1 ± 1.2	0.107	0.08
Network	0.5 ± 1.6	−0.7 ± 1.6	−0.3 ± 1.5	0.4 ± 2.0	0.1 ± 1.8	0.0 ± 1.6	0.820	0.175
Social participation	0.0 ± 1.0	0.3 ± 1.2	0.3 ± 1.0	0.4 ± 0.8	−0.3 ± 0.8	−0.2 ± 0.9	− 0.846	0.839

Values are expressed as the mean ± SD ^**^

simple main effect compared to pre intervention, *p* < 0.01

## Discussion

The results of this study suggest that the combined effects of social network incentives and financial incentives continued after the intervention and increased physical activity in older women, but those receiving only financial incentives were not as effective in this study.

Aharony et al. [21] focused on social network incentives leveraging the power of peer pressure by using peer rewards. In their experiments, the process by which the target participants’ good behavior rewards the “buddy” enhanced the physical activity of both the target and the “buddy.” Furthermore, the study found that increased physical activity continues after the intervention has ended when financial incentives are combined with social network incentives. Therefore, in our study, although there was no effect during the intervention period, we expected the effect on the increase in physical activity to continue after the intervention. In addition, the results of our study are consistent with those of Aharony et al. However, a key distinction of our study is that it is the first to investigate whether social network incentives have beneficial effects on physical activity among older persons after an intervention using a randomized study.

In a previous study about the effects of financial incentives alone, it was found that even when tangible rewards were offered, they decreased intrinsic motivation for activity [18]. Additionally, the rewards are likely to be accompanied by surveillance, evaluation, and competition, which have also been found to undermine intrinsic motivation [33]. For these reasons, our study concluded that financial incentives alone did not increase physical activity during the intervention period. Conversely, if the rewards were higher, physical activity might have increased. High financial incentives, with total possible rewards exceeding $100, have been found to create change in behavior [34, 35]. However, once the incentives are no longer apparent, lasting effects have been observed only in a few cases. Therefore, it is interesting that by using social network incentives, even low financial incentives have beneficial effects on physical activity in older persons, even after an intervention. Additionally, in older persons, maintaining increased physical activity will not only prevent cardiovascular disease and improve QOL [1–4, 6, 7] but also prevent cognitive function and depression [8–11], which may prevent receipt of care.

In our research, we examined whether physical activity interventions increased individual-level social capital, however, we were unable to prove our hypothesis. Several previous studies have shown that increasing individual-level social capital is not easy. In their study on social capital intervention, Moore et al. summarized that it is important to increase network diversity [36]. In addition, Ottesen et al. [37] conducted a focus group interview on team sports to enhance social capital. Furthermore, we used the I-, we- and they-stories as a technique for building social capital. Based on these previous studies, we considered that further intervention is necessary in this study. For example, we may have enhanced social capital by creating opportunities for participants in this study to interact with large groups and approach individuals.

Putnam and Feldstein’s study [38] showed that smaller groups with face-to-face communication promote empathy about the factors that create social capital among the participants. In our study, we formed groups of three people to create the power of peer pressure. We believe that groups of three people are the minimum size necessary to maximize social network incentives and that even numbers have the risk of causing division. Additionally, previous studies have shown an association between individual-level social capital, and self-rated physical and mental health [39–41]. Therefore, increasing the number of people walking with buddies in the region may contribute to the development of local social capital and health.

There were a few limitations to this study. First, the sample was small, with females only, and we limited the study to Japan’s regions only. Additionally, because our sample was healthy, the results may not be generalizable to less healthy people, men, and other racial or ethnic groups. Future studies should involve a large number of participants and men. Second, although a randomized design was employed, the study enrolled only those persons who voluntarily responded to an advertisement and agreed to participate in the program. Third, the researchers only collated the pedometer and diary data for 1 week, and not the data beyond that timeframe. Moreover, because physical activity was measured using a pedometer, we could not quantify other forms of physical activity, such as cycling or swimming. Finally, it is unclear whether different incentive designs would yield greater effectiveness at a lower cost.

## Conclusion

Our results suggest that social network incentives combined with financial incentives are more effective for promoting physical activity among older women than financial incentives alone and that these effects can continue postintervention. We therefore recommend a sustainable walking program that can lead to better health for older persons. In the meantime, further studies should be conducted on the effect of physical activity on social capital.

## Data Availability

The datasets used and/or analyzed during the current study are available from the corresponding author on request.

## Abbreviations

FI: financial incentive
SNI: social network incentive
ANOVA: analysis of variance
